# A Supraparticle‐Based Approach to Robust Biomimetic Superhydrophobic Coatings

**DOI:** 10.1002/smll.202505850

**Published:** 2025-09-05

**Authors:** Umair Sultan, Teresa Walter, Christian Wachter, Leon Swart, Nicolas Vogel

**Affiliations:** ^1^ Institute of Interfaces and Particle Technology Friedrich‐Alexander‐Universität Erlangen‐Nürnberg Cauerstrasse 4 91058 Erlangen Germany

**Keywords:** biomimetic, repellent coatings, robustness, superhydrophobic, supraparticles

## Abstract

Repellent surfaces provide resistance to biofouling, ice formation, bacteria adhesion, or corrosion. Inspired by the hierarchical structure of the lotus leaf, such surfaces minimize water adhesion through micro‐ and nanostructuring. Conventional fabrication methods to mimic the lotus leaf often involve problematic fluorinated compounds, sophisticated preparation conditions, or lack mechanical robustness. This study presents a fluorine‐free, scalable approach for fabricating durable superhydrophobic coatings using supraparticles. Supraparticles are structured aggregates of colloidal primary particles and serve as intermediate building blocks that provide hierarchical surface roughness. These supraparticles are fabricated by spray drying and introduce hydrophobic surface properties via alkyl‐silanes. These preassembled structures are then simply spray coated onto a polymeric primer layer to create a coating with hierarchical roughness features, mimicking the surface of the lotus leaf. The performance of the coatings is assessed by water contact angles, contact angle hystereses, roll‐off angles, and water droplet pinning fraction, and shows how the robustness can be enhanced by the addition of binder and choice of primer layer. The method offers an experimentally simple, scalable, and versatile process strategy for robust superhydrophobic coatings.

## Graphical abstract

Superhydrophobic coatings are fabricated using supraparticles as intermediate building blocks with inherent hierarchical roughness features. Applied via spray‐coating onto a polymeric primer to anchor the supraparticles, these coatings show a hierarchical micro‐nanostructure, closely mimicking the lotus leaf. High durability is achieved by enhancing the mechanical properties of both supraparticles and the primer layer.

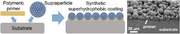

## 1 Introduction

**Figure 1:**
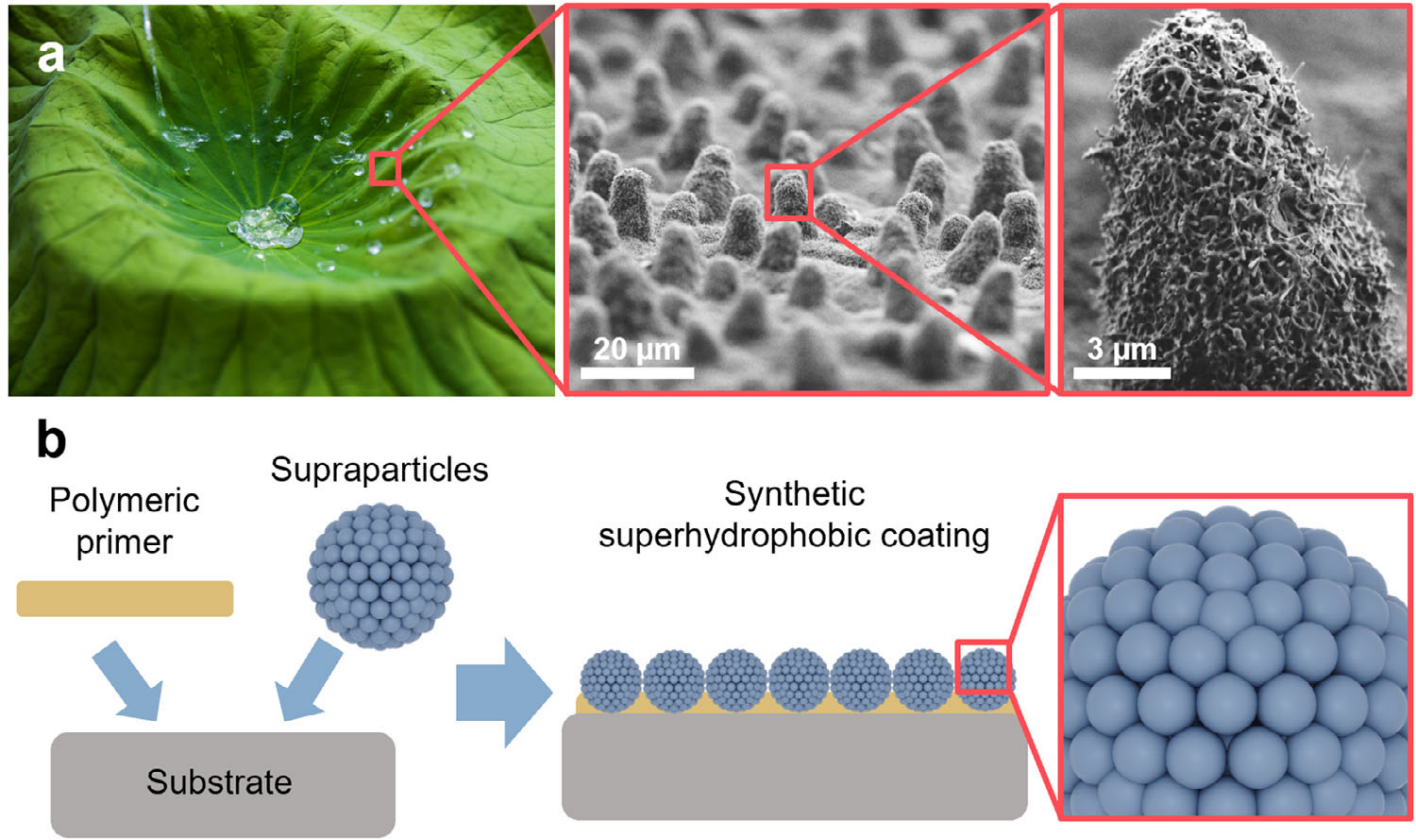
Superhydrophobic repellent surfaces. a) Photograph and SEM images of the lotus leaf surface structure showing the microstructure formed by the papillae and the nanostructure formed by epicuticular wax crystals. b) Schematic illustration of the synthetic supraparticle‐based coating with hierarchical topography.

Repellent, self‐cleaning coatings prevent undesired surface contamination and find applications in the prevention of biofouling,^[^ ^1^, ^2^, ^3^, ^4^ ^]^ anti‐icing,^[^ ^5^, ^6^, ^7^ ^]^ corrosion protection,^[^ ^8^, ^9^, ^10^ ^]^ and cell culture and patterning.^[^ ^11^, ^12^ ^]^ A key inspiration for artificial superhydrophobic surfaces is the lotus leaf (*Nelumbo nucifera*),^[^ ^13^ ^]^ which exhibits remarkable water repellency due to its unique hierarchical surface structure combined with hydrophobic surface chemistry.^[^ ^14^ ^]^ Its surface consists of small papillae, which have a size of around 2–14 µm, and a spacing of 5–20 µm, as shown in **Figure** **1**a.^[^ ^15^ ^]^ These papillae are further covered with epicuticular wax crystals (Figure 1a), which exhibit a tubular structure with a thickness of 80–120 nm.^[^ ^16^, ^17^, ^18^ ^]^ This combination of nanostructures on top of a microstructure minimizes the contact area and adhesion of water droplets,^[^ ^19^ ^]^ resulting in the well‐known Cassie‐Baxter wetting state, which provides superhydrophobicity with effective water repellency.^[^ ^20^ ^]^

To replicate this surface roughness, several fabrication methods have been developed, including nanoimprint lithography,^[^ ^21^, ^22^ ^]^ reactive ion etching,^[^ ^23^, ^24^ ^]^ 3D printing,^[^ ^25^, ^26^, ^27^ ^]^ layer‐by‐layer assembly,^[^ ^28^, ^29^, ^30^ ^]^ in situ polymerization,^[^ ^31^ ^]^ wire rod coating,^[^ ^32^ ^]^ electrophoretic deposition,^[^ ^33^ ^]^ or chemical etching.^[^ ^34^, ^35^ ^]^ While effective, these techniques typically require specialized infrastructure, equipment, or materials. Furthermore, many strategies to induce repellency rely on organic solvents or fluorinated compounds,^[^ ^36^, ^37^, ^38^, ^39^, ^40^, ^41^ ^]^ which are prone to bioaccumulation and are persistent in the environment.^[^ ^42^, ^43^, ^44^ ^]^ Beyond sustainability and scalability, mechanical durability is another critical challenge for superhydrophobic surfaces, as the nanoscale features required for efficient repellency are prone to mechanical damage.^[^ ^41^, ^45^, ^46^ ^]^

Further advancements of such coatings, therefore, need to focus on simple and environmentally benign coating strategies, and approaches to stabilize roughness features at the smallest scale against mechanical damage. Recent studies have, therefore, also focused on the durability of superhydrophobic coatings.^[^ ^47^, ^48^, ^49^ ^]^

Here, we propose to address these challenges using supraparticles (SPs), which are defined aggregates of smaller colloidal building blocks called primary particles.^[^ ^50^, ^51^ ^]^ These SPs act as intermediate building blocks to introduce the hierarchical surface topography that mimics the structure of the lotus leaf (Figure 1b). The idea behind this concept is that the SPs provide a preformed, microscopic structure that already contains the desired nanoscale roughness features from the primary particle building blocks. These scalable intermediates can then be handled as a conventional powder and be coated onto a surface to create a defined, hierarchical topography in a simple process step. Therefore, the SP‐based approach facilitates the creation of the required hierarchical roughness features, which otherwise often involve complex surface patterning or careful control of drying processes. In addition, the individual primary particles providing the nanoscale roughness features are firmly anchored within the SP, and their integration can be further enforced via the addition of binders, which enhances the mechanical stability of the entire coating.

SPs can be formed by the confined assembly of the primary particles in a liquid droplet either by emulsification,^[^ ^52^ ^]^ on superhydrophobic surfaces,^[^ ^53^, ^54^ ^]^ or spray drying.^[^ ^55^ ^]^ This assembly gives rise to inherent emergent properties due to the ordered arrangement of primary particle within SPs, such as structural coloration,^[^ ^56^ ^]^ hierarchical porosity,^[^ ^57^ ^]^ or defined surface roughness.^[^ ^58^ ^]^ In our case, we take advantage of the surface roughness of SPs to fabricate robust repellent surfaces.

To fabricate superhydrophobic coatings, we first engineer the SPs to optimize performance by controlling powder size distributions, molecular surface functionality, and mechanical properties. The SPs are subsequently deposited onto the substrate with a polymeric primer layer by spray coating, ensuring uniform distribution and adhesion with the primer layer (Figure 1b). We evaluate their performance by measuring the water contact angle, contact angle hysteresis, roll‐off angle, and water droplet pinning fraction. Finally, we assess the mechanical robustness of the resulting water‐repellent coatings to determine their durability under various conditions.

## 2 Results and Discussion

**Figure 2:**
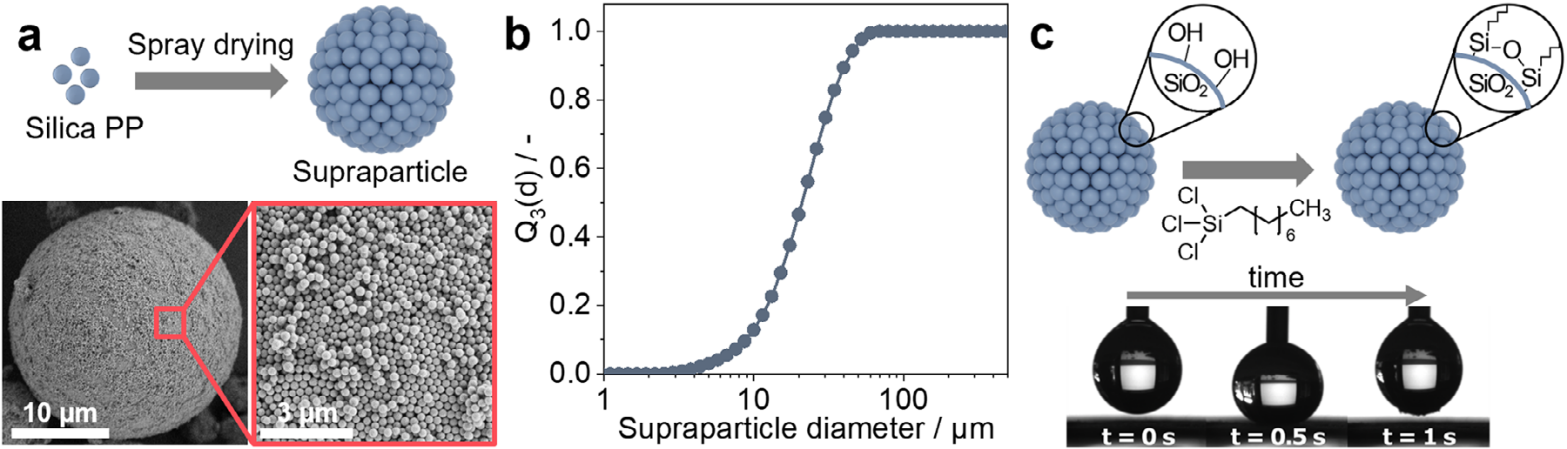
Fabrication of supraparticles with defined nanoscale surface topography. a) Schematic illustration and SEM images of the supraparticle and its rough surface. b) Particle size distribution of the spray‐dried supraparticles. c) Schematic illustration of the surface modification of supraparticles using octyl‐trichlorosilane and time‐lapsed photographs of a water droplet on the surface‐modified supraparticles.

We start by fabricating SPs with defined surface topography. To this end, we synthesize silica colloidal primary particles with an average diameter of ≈200 nm using the Stöber method (Figure S1, Supporting Information).^[^ ^59^ ^]^ These primary particles are then assembled into SPs via spray drying.^[^ ^60^ ^]^ The fabricated SPs exhibit a well‐defined structure and spherical shape, as shown in **Figure** **2**a (Figure S2, Supporting Information). The surface of the SP is composed of close‐packed primary particles, forming a defined surface roughness. We define the diameter of the SP by the circumference where the outermost layer of primary particles are in close contact. This allows us to define the dimension of the surface roughness, which corresponds to the height of the primary particles protruding from the SP surface, i.e., their radius (Figure S2c, Supporting Information). Since this surface roughness scales directly with the size of the primary particles,^[^ ^61^ ^]^ we select a diameter of 200 nm for the primary particles to obtain a surface roughness of 100 nm, which mimics the dimensions of the epicuticular wax crystals on the papillae of the lotus leaf, forming the nanoscale roughness.^[^ ^17^ ^]^ This surface topography is evident from the SEM images shown in Figure 2a, where the arrangement of primary particles on SP surface can be directly observed. The SP powder is fabricated to have an average diameter of ≈15 µm (Figure 2b) to mimic the size range of the papillae that form the micronscale roughness of the hierarchical surface structure of the lotus leaf.^[^ ^15^ ^]^ In its native form, the surface chemistry of Stöber silica is composed of silanol groups that are hydrophilic in nature.^[^ ^62^ ^]^ We therefore chemically modify their surface with hydrocarbon chains by silanization with octyl‐trichlorosilane in vacuum to avoid the use of organic solvents in the process. The resulting SP powder is hydrophobic, as is evident from the series of images in Figure 2c where a droplet of water fails to wet the SP powder bed.

We then fabricate superhydrophobic coatings using the SPs as intermediate building blocks that already contain the desired hierarchical surface structure. To achieve this, we use a glass slide as a model substrate and coat this substrate with a thin polymeric primer whose function is to anchor the SPs to the substrate. We start by using polydimethylsiloxane (PDMS) as the primer layer, which forms a sticky film upon application that subsequently hardens by a hydrosilylation reaction to form an elastic polymer film. The functionalized SPs are dispersed in ethanol and spray coated onto the primer layer (experimental details in Method section). The coating is then cured in the oven at 80 °C to harden the primer layer. Note that the adhesion of the supraparticles to the coating is mediated by the viscous nature of the polymer primer layer during the spray coating, and not by using a solvent to partially dissolve the base layer. Ethanol was selected for the spray coating due to its compatibility with our existing laboratory setup and its ability to effectively disperse hydrophobic supraparticles. Additionally, ethanol is a more environmentally benign option compared to other organic solvents.

**Figure 3:**
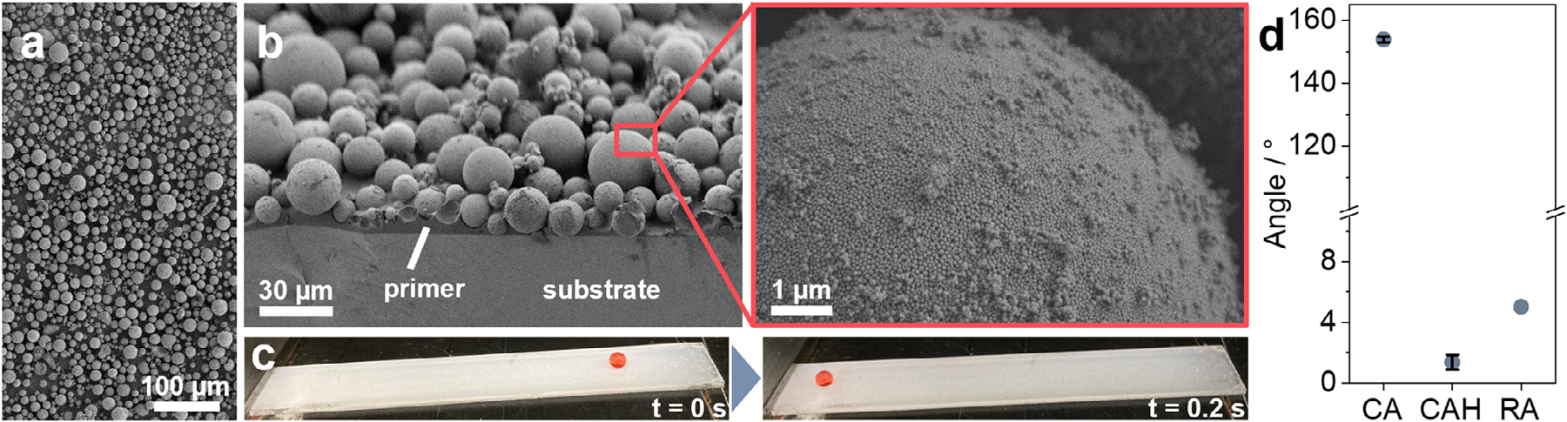
Superhydrophobic coatings from supraparticles. a)Top‐view SEM image of the fabricated supraparticle‐based repellent coating. b) Side‐view SEM image of the fabricated coating showing the polydimethylsiloxane (PDMS) primer layer, the supraparticles embedded in the primer layer, and the supraparticle surface. c) Time‐lapsed photographs of a dyed water droplet rolling off the fabricated repellent coating. d) Static water contact angle (CA), contact angle hysteresis (CAH), roll‐off angle (RA) of the repellent coatings.

The resulting coatings show a high coverage with a single cycle of spray coating (**Figure** **3**a); additional spray cycles do not significantly increase the surface coverage with SPs (Figure S3, Supporting Information). A representative SEM image of the coating cross‐section (Figure 3b), taken at an angle of 60°, shows the topography of the coating, consisting of SPs embedded within the polymeric primer layer on the glass substrate. The SPs form a microstructure resembling the papillae on the lotus leaf (Figure 1a), while the primary particles within the SPs mimic the nanostructure formed by the epicuticular wax crystals. This hierarchical micro‐ and nanostructure imparts the coating with superhydrophobic properties, demonstrated by the time‐lapsed photos in Figure 3c that show a droplet of dyed water rolling off the coated surface at a tilt angle of 5°. Video S1 (Supporting Information) further illustrates this effect with an ink droplet rolling down the fabricated superhydrophobic surface. We quantify the wetting properties of the produced coating through water contact angle analysis, which shows a static contact angle (CA) of 154° ± 1.3, a low contact angle hysteresis (CAH) of 2° ± 0.6, and a roll‐off angle (RA) of 5°, confirming the water repellent properties.^[^ ^63^ ^]^ Note that dip‐coating can also be used and lead to coatings with similar repellent properties (Figure S4, Supporting Information).

**Figure 4:**
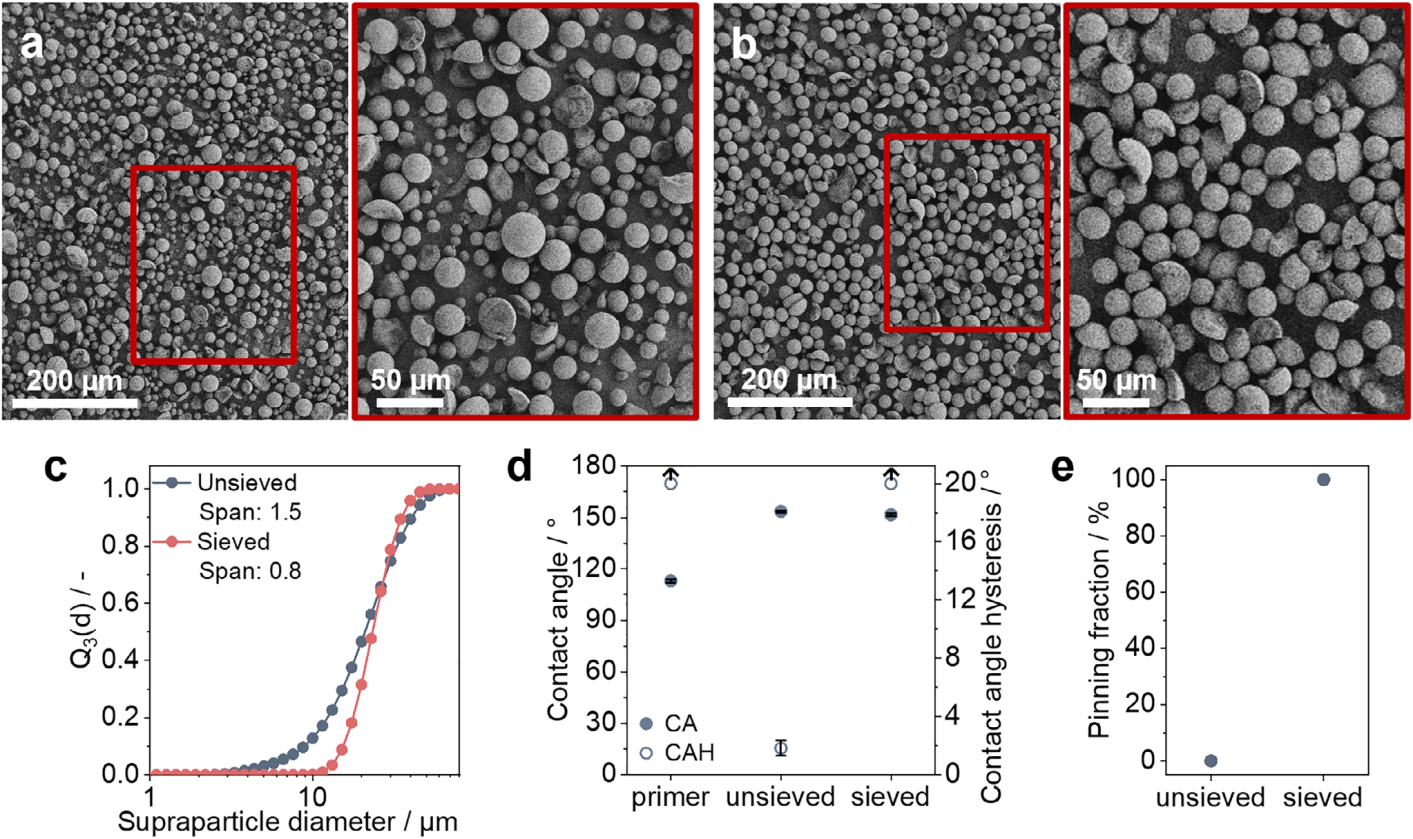
Superhydrophobic coatings using SPs with different size distributions a,b) Top‐view SEM images of the coatings fabricated from unsieved and sieved supraparticles, respectively. Insets show the magnified view of the coatings. c) Particle size distributions of unsieved and sieved supraparticles. d) Static contact angle (CA) and contact angle hysteresis (CAH) of the polydimethylsiloxane (PDMS) primer layer and the coatings fabricated using unsieved and sieved supraparticles. The arrows on hollow circles indicate a very high CAH due to droplet pinning. e) Pinning fraction (from 10 water droplets) of each of the fabricated coatings.

As‐fabricated spray dried SPs exhibit a broad size distribution,^[^ ^52^ ^]^ prompting us to investigate the influence of SP size distribution on the performance of superhydrophobic coatings. To achieve a narrower size distribution, we separate the spray dried SPs using wire mesh sieves of 20 and 25 µm, resulting in a size distribution with a span of 0.8 (**Figure** **4**b), which is half that of the as‐fabricated SPs' original span of 1.5. We then use these sieved SPs to prepare superhydrophobic coatings, following the procedure described above. The difference in the particle size distribution is clearly visible in the top‐view SEM images of the fabricated coatings in Figure 4a,b. Water contact angles of both types of coatings, shown in Figure 4d, reveal that the bare PDMS primer layer is hydrophobic (CA = 113° ± 0.9), but has a very high hysteresis (106° ± 5.3) due to droplet pinning, which is indicative of poor water repellency properties. After the incorporation of the SP layer onto the primer layer, the contact angle increases significantly. For both sieved and unsieved coatings, we find CAs>150°. Surprisingly, despite similar apparent superhydrophobic properties, the CAH of both coatings differs significantly. The coating from unsieved SPs has a CAH of <3° and thus, exhibits efficient water repellency properties. In contrast, the coating formed by sieved SPs shows a very high CAH (Figure 4d). We further compare the ability of the coatings to repel water droplets by determining the fraction of pinned droplets on coatings tilted at a 5° angle (Figure 4e). Coatings prepared with unsieved SPs do not show pinning, while those with sieved SPs exhibit a 100% pinning fraction, indicating poor repellency.

This behavior is surprising at first glance because one would assume that the more uniform nature of the sieved particles, which allows them to form a more homogeneous SP coating, would enhance their repellent properties. However, if we closely observe the SEM images of the two coatings shown in Figure 4b,c, the broader size distribution of the unsieved SPs creates a microstructure where the smaller SPs surround the larger ones that are spaced apart, similar to how the papillae in the lotus leaf are arranged (Figure S5, Supporting Information). We hypothesize that this provides a larger height difference due to the differently‐sized SPs, i.e., a higher microscale roughness and prevents droplet pinning by increasing the relative distance between the bottom of a droplet and the point of contact between adjacent SPs. In contrast, the sieved SPs with similar sizes in close contact provide a smaller height difference between the top of the SPs and their point of contact. This leads to a relatively shorter distance between the wetting droplet and the contact point between the SPs. This may facilitate attachment of the droplet, potentially by creating a Wenzel wetting state at the micro‐level. This is analogous to the rose petal effect, where the droplet is also pinned by the micro‐level structure (Wenzel state), but due to the additional presence of a nanostructure still exhibits a high contact angle (Cassie‐Baxter state).^[^ ^64^, ^65^ ^]^ Thus, a seemingly subtle difference in SP size distribution makes a surprising difference in terms of their repellency properties. Importantly, this finding simplifies the coating process since the as‐fabricated SPs can be directly used to produce efficient superhydrophobic coatings without requiring additional processing steps to tailor the size distribution.

**Figure 5:**
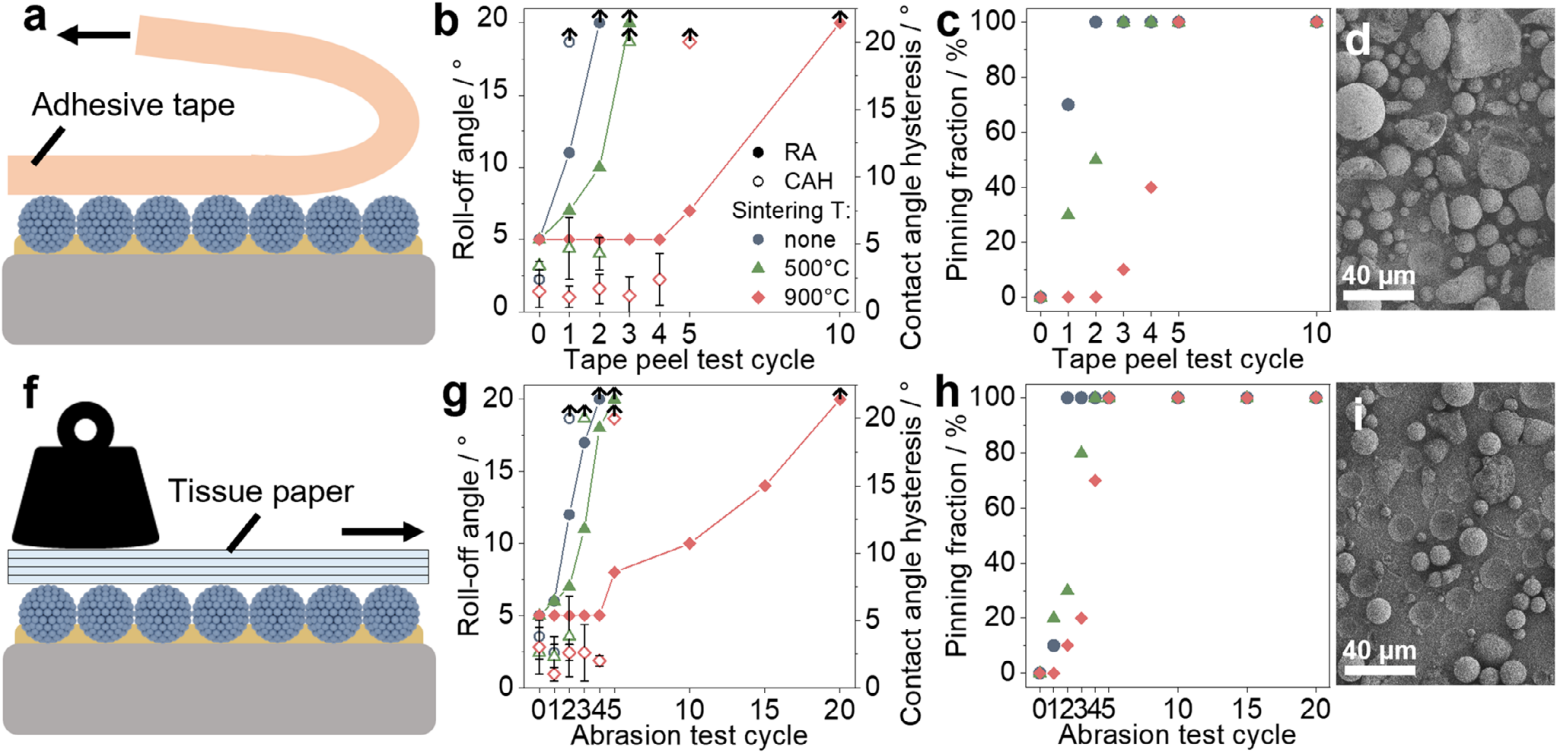
Mechanical stability of coatings prepared with a polydimethylsiloxane (PDMS) primer layer and unsintered and sintered supraparticles. a,f) Schematic illustration of a) tape test and f) abrasion test of the fabricated coatings. b,g) Roll‐off angle (RA) and contact angle hysteresis (CAH) of the coatings subjected to the b) tape peel test and g) abrasion test. c,h) Pinning fraction (from 10 water droplets) of the coatings after c) tape peel test and h) abrasion test cycles. d) SEM image of coatings made from unsintered supraparticles after one cycle of tape peel test (point of failure). i) SEM image of coatings made from supraparticles sintered at 900 °C after five cycles of tape peel test (point of failure).

Biomimetic, lotus leaf‐like coatings are prone to mechanical failure due to damage of their nanoscale roughness features.^[^ ^45^, ^46^ ^]^ We hypothesize that using SPs, where the individual primary particles are locked in a preformed assembly, will improve their stability, and thus the mechanical robustness of the entire coating. We assess the mechanical stability of our coatings using two methods: a tape test and a linear abrasion test. The as‐fabricated SPs are held together only by comparatively weak contact forces. To increase their mechanical strength, we sinter them at high temperatures (500 and 900 °C) to form solid bridges connecting the individual primary particles (**Figure** **5**).

The tape test evaluates SP adhesion by applying a scotch tape to the coating and peeling it off horizontally, as shown in Figure 5a. The non‐sintered SPs lose their repellency after just one test cycle, whereas SPs sintered at 500 °C withstand two cycles before failure, as evidenced by large CAHs and a high pinning fraction (Figure 5b,c). In these cases, the coating failure primarily results from SP breakage, indicating that the mechanical strength of the SPs themselves is the limiting factor (Figure 5d). When the SPs are calcined at 900 °C, their mechanical properties are enhanced due to the formation of silicon‐oxygen‐silicon bonds between the primary particles via condensation of surface silanol groups. The resultant coatings retain their repellency through 5 cycles of tape test before failing. We attribute this failure to an adhesive failure caused by insufficient adhesion between SPs and the PDMS primer layer,^[^ ^66^ ^]^ causing the complete detachment of SPs, as indicated by the impressions left in the primer layer, which can be observed in the SEM image in Figure 5i.

The linear abrasion test is conducted to assess the overall integrity of the coatings by placing a folded tissue paper on the coating, applying 100 g of weight on top, and pulling the tissue paper over the coatings, as illustrated by Figure 5f. The results of the abrasion test corroborate the data from tape tests. Coatings from non‐sintered SPs exhibit the lowest mechanical stability, losing their repellency after two test cycles, while SP sintered at 500 °C lose repellency after three cycles (Figure 5g). The highest stability is observed in coatings with SPs sintered at 900 °C, which fail after 5 test cycles, indicated by large CAH, increased RA, and a larger pinning fraction (Figure 5g,h). For all cases, CA values only show minimal change after multiple cycles of testing (Figure S6, Supporting Information).

**Figure 6:**
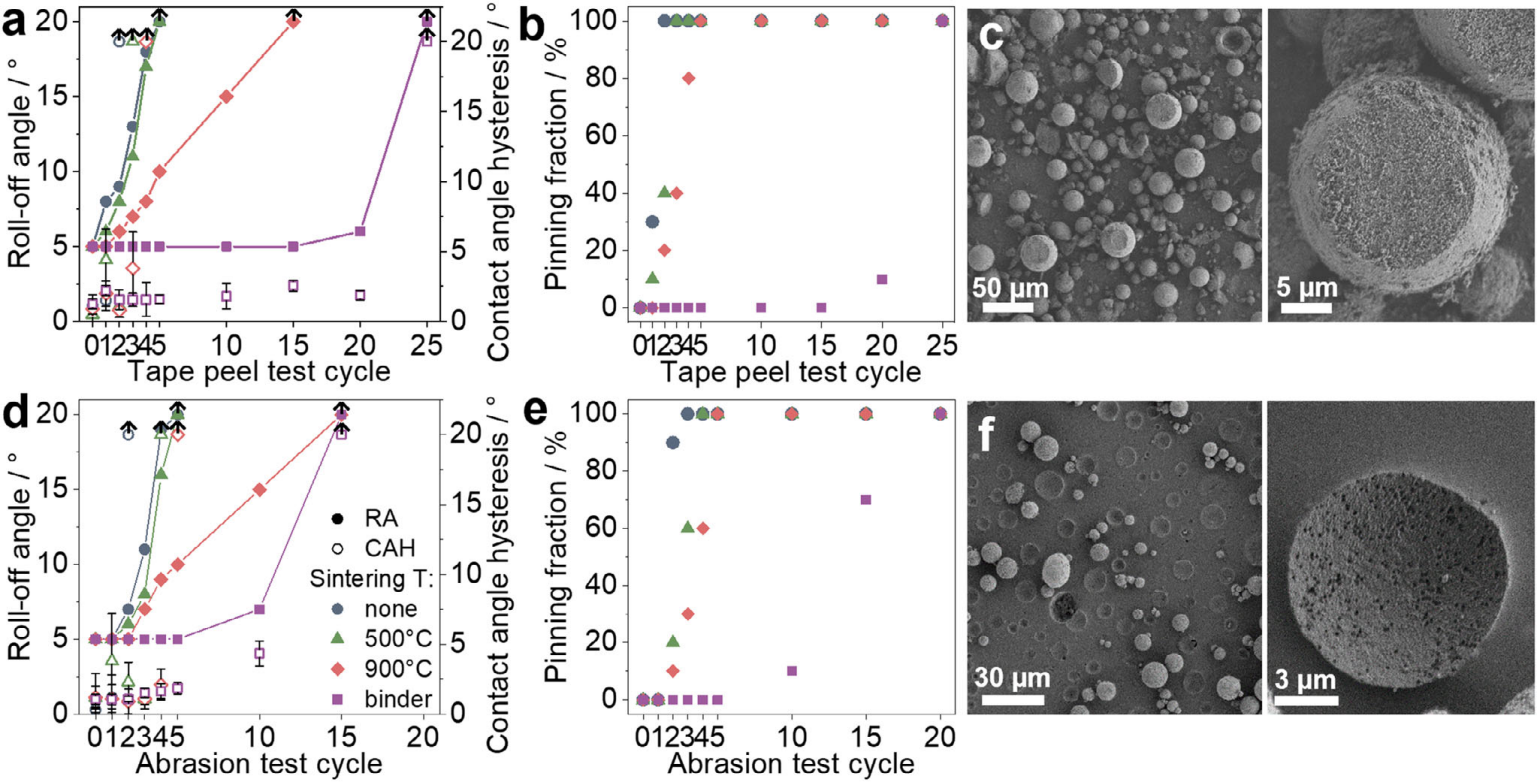
Mechanical stability of coatings made using polyurethane (PUR) primer layer and unsintered and sintered supraparticles. a,d) Roll‐off angle (RA) and contact angle hysteresis (CAH) of the coatings subjected to the a) tape peel test and d) abrasion test. b,e) Pinning fraction (from 10 water droplets) of the coatings after b) tape peel test and e) abrasion test cycles. c) SEM image of coatings made from supraparticles sintered at 900 °C after 15 cycles of abrasion test (point of failure). f) SEM image of coatings made from supraparticles sintered, containing water glass binder, after 25 cycles of tape test (point of failure).

To enhance the mechanical stability of the coatings and address the SP adhesion issue observed in coatings fabricated using a PDMS primer, we employ polyurethane (PUR) as a more robust primer layer.^[^ ^67^ ^]^ Coatings fabricated using PUR exhibit similar superhydrophobicity and water repellent properties as the PDMS‐based coatings (Figure S7, Supporting Information). Upon evaluating the mechanical stability of coatings fabricated with a PUR primer layer (**Figure** **6**), we observe a trend similar to that of PDMS‐based coatings. In both tape and abrasion tests, non‐sintered SP coatings fail after two test cycles, followed by coatings with SPs sintered at 500 °C. As the failure of these coatings results from SP breakage, it is independent of the type of primer layer. However, coatings with SPs sintered at 900 °C retain their repellency for up to 4 cycles of the tape test and 5 cycles of the abrasion test. This time, their failure is not due to adhesion issues but rather the structural stability of the SPs themselves. As evidenced in Figure 6c, after 5 abrasion cycles, the SPs undergo mechanical grinding and breakage but remain embedded in the PUR primer.

To counteract the SP breakage, we introduce water glass as a binder during spray drying to reinforce the contact points between the primary particles. The SPs are then sintered at 900 °C to ensure complete condensation of the water glass. These strengthened SPs maintain similar surface roughness characteristics and particle size distribution as their binder‐free counterparts (Figure S8, Supporting Information). Coatings prepared using these optimized SPs, in combination with PUR as a primer, demonstrate significantly enhanced mechanical stability, as shown in Figure 6d,e. These optimized coatings withstand up to 25 cycles of the tape test and 15 cycles of the abrasion test before losing their repellency, as indicated by their consistently low CAH and RA values. Pinning fraction analysis further corroborates the mechanical robustness, showing minimal pinning even after multiple test cycles. The CA remains large with minimal changes after the mechanical tests (Figure S9, Supporting Information). A summary of the mechanical stability results for various coatings is provided in Table S1 (Supporting Information). SEM images of the coating after 25 cycles of tape peel test (Figure 6f) reveal that while the SPs remain intact, coating failure occurs due to SP detachment from the PUR primer. Therefore, further improvements in mechanical stability would require further enhancing the mechanical properties of the primer layer, possibly by crosslinking, or the use of epoxy‐based resins.

We further demonstrate the robustness of the coatings by subjecting them to impinging streams and droplets of both pure water and acidic solution (pH 1). As evidenced by Videos S2 (water) and S3 (pH 1) (Supporting Information), the coatings remain repellent after the tests. Wetting data presented in Figure S10 (Supporting Information) confirm that the coatings maintain their repellency, i.e., low CAH and RA, even after exposure to highly acidic (pH 1) and highly basic (pH 14) liquids. Finally, we tested the self‐cleaning performance of the coatings by spreading fluorescent polymeric particles on it, simulating solid impurities. These particles were easily removed by washing with water, as demonstrated in Video S4 (Supporting Information).

## 3 Conclusion

We develop a simple, scalable method for fabricating robust superhydrophobic coatings inspired by the surface of the lotus leaf. Our strategy is based on spray dried SPs that act as preformed, intermediate building blocks that already contain the desired hierarchical surface structure and hydrophobic surface chemistry. The coating is formed by a single spray coating step and anchored to the substrate using a polymeric primer. Our coatings exhibit efficient water repellent properties, evidenced by high contact angles (>150°), low contact angle hysteresis (<3°), low roll‐off angles (5°), and no droplet pinning. These properties are consistently maintained across different primer layers. We find that as‐fabricated SPs produce coatings with superior repellency compared to more uniform, sieved SPs due to the formation of a more pronounced micro‐scale roughness. Mechanical stability tests reveal that sintering SPs at 900 °C significantly improves their durability by avoiding cohesive failure within the SPs themselves, while using PUR as a primer layer further suppresses adhesive failure by enhancing SP adhesion to the substrate. Introducing water glass as a binder further reinforces the SP structure, leading to highly durable coatings that withstand up to 25 cycles of tape peel test and 15 abrasion cycles. The structure‐property relations connecting mechanical robustness with process conditions to strengthen inter‐supraparticle bonds and thus hinder cohesive failure, and the choice of primer layer to avoid adhesive failure, provide general guidelines to further enhance the mechanical properties of the coating. More sophisticated surface chemistries with the potential to form covalent bonds between the primary particles, thus further strengthening SP cohesion. A different choice of the primer layer may further provide covalent bonds to the SP surface, which may further strengthen SP adhesion to the primer layer. Our findings highlight the potential of using SPs as convenient intermediates to form lotus leaf‐like repellent surfaces using simple and scalable processes without requiring the use of problematic organic solvents. We optimize robustness by identifying and systematically enhancing the weakest interface responsible for failure. Our approach thus offers a promising route toward environmentally benign solutions that combine superhydrophobic functionality with simple processing and long‐term durability.

## 4 Experimental Section

### Synthesis of Silica Primary Particles

Silica primary particles with 200 nm diameter were synthesized using a modified protocol from literature.^[^ ^68^ ^]^ 8 m water (Milli Q water, 18.2 mΩ) was mixed with 0.18 m ammonia (32%, pure, Carl Roth), 0.3 m TEOS (≥99%, Sigma–Aldrich), and filled up to 1500 mL with absolute ethanol (≥99.8%, puriss., p.a., absolute, Sigma–Aldrich). The solution was left to stir for 16 h at room temperature and a stirring rate (magnetic bars ROTILABO elliptical, ∅: 20 mm, 50 mm) of 350 rpm. The particles were purified three times by centrifugation and redispersion in a 50:50 water‐ethanol mixture and then twice by centrifugation and redispersion in water. Finally, the primary particles were redispersed in water to a concentration of 40 wt.%.

### Fabrication of Supraparticles

The SPs were fabricated as described before in the literature.^[^ ^57^ ^]^ Using a spray dryer (B‐290 Mini Spray Dryer by Büchi) the primary particle‐water‐dispersion was dried under a nitrogen atmosphere. A co‐current flow two‐fluid nozzle with a nozzle size of 1.4 mm was used. The inlet temperature was set to 120 °C. The atomization gas flow was set to 357 L h^−1^, and the feed flow rate of the colloid was kept at 3 mL min^−1^. The aspirator was set to a flow rate of 35 m^3^ h^−1^. The fabricated SPs were either used directly or sintered using a furnace (M110 Laboratory Muffel Furnace, Thermo Scientific) for 7 h at 500 and 900 °C. For the binder containing SPs, water glass (extra pure, Sigma Aldrich) was added to the colloid (0.1 wt% of the solid content) before spray drying. Afterward, the binder‐containing SPs are sintered at 900 °C for 7h. The size distribution of the SPs was reduced by sieving them between wire mesh sieves of size 25 and 20 µm.

### Surface Modification of Supraparticles

The surface of the SPs was chemically modified via silanization in vapor phase. Before the silanization process, the SPs were treated in a plasma oven (Femto, Diener electric). For this, they were placed in a glass container covered with a wire mesh to prevent them from spreading in the oven. The treatment was carried out for 10 min under an oxygen‐atmosphere with a gas flow of 5 L min^−1^ at 100 Watt and 0.02 mbar. The SPs were then placed together with 500 µL of octyl‐trichlorosilane (97%, Sigma Aldrich) in a desiccator and pulled a vacuum up to 0.1 mbar, after which the vacuum pump was shut down and the samples were left in the desiccator for 24 h. Afterward, they were thermally treated at 110 °C for 1 h to ensure complete condensation of the silanes.

### Preparation of Substrates

Glass microscope slides (Menzel‐Gläser by Thermo Scientific, ISO 8037/1, 26 mm x 76 mm) were used as a substrate for the superhydrophobic coatings. To remove impurities, the glass slides were cleaned for 5 min in an ultrasonic bath first in acetone, then in ethanol, and finally in water. Afterward, the glass slides were dried with compressed air and cleaned with lint‐free Kimwipe tissues (KIMTECH Science precision wipes by Kimberly Clark, type 7552) mechanically by hand.

### Preparation of Primary Layer

Polydimethylsiloxane (PDMS) was used as a hydrophobic primer layer. PDMS consists of two components. The silicone elastomer (DOWSIL 184 Silicone Elastomer Base) and the hardener (DOWSIL 184 Silicone Elastomer Curing Agent) were mixed in a ratio of 10:1 and stirred very well by hand. Additionally, polyurethane (PUR) was used as a hard primer layer. For this, 2,2,4‐trimethylpentan‐1,3‐diol and 2‐butyl‐2‐ethylpropandiol (SuK Hock GmbH) as the casting resin was mixed with the hardener hexamethylen‐1,6‐diisocyanat (SuK Hock GmbH) in a ratio of 1:1.5, as specified by the supplier, and 4 wt.% (3‐aminopropyl)‐triethoxysilane (APTES) to improve substrate wettability.^[^ ^69^ ^]^

The primary layer was applied via spin coating (WS‐650MZ‐23NPPB, Laurell). All spinning cycles were run for 30 s with an acceleration speed of 1000 rpm s^−2^. 0.15 g of the polymer was used. The spinning speed was 8000 rpm for PDMS and 3000 rpm for PUR.

### Preparation of Superhydrophobic Coatings

The SPs were applied via spray coating to the primary by spraying a 5 wt.% SP‐ethanol dispersion using a spray gun (Evolution Airbrush, Harder & Steenbeck) with the sample fixed at a distance of 15 cm. The nozzle size was 400 µm, and an air pressure of 3 bar was applied. A horizontal velocity of 5 mms^−1^ and a flow rate related to water of 133.2 µLs^−1^ were used for spray coating. Before the application of SPs, the primer coatings were pre‐cured inside an oven at 80 °C for a time of 2 min for PDMS and 15 min for PUR to ensure optimal viscosity and thus, effective embedding of SPs inside the primer. Shorter times lead to the SPs being coated completely by the primer and losing superhydrophobicity, while longer times lead to superficial attachment of the SPs. After the application of SPs, the substrates were placed back inside an oven at 80 °C for 4 h for PDMS and at least 24 h for PUR to achieve complete curing. After curing, all the coatings were thoroughly washed with ethanol and water to remove any residuals and contaminations.

### Characterization of Surface Wetting and Repellent Properties

Contact angle and contact angle hysteresis were measured using a Krüss DSA 100. All analysis were done using the tangent method. For static contact angle and contact angle hysteresis measurements, 5 µL water droplets were used. Pinning fraction and roll‐off angles were measured on a tilted stage by using water droplets of 10 µL. For measuring the roll‐off angle, three water droplets were placed on the coating, with an initial angle of 5°, the sample was then tilted more and more. The roll‐off angle is the angle at which all the droplets moved over the surface. The pinning fraction was determined by fixing the sample at a tilting angle of 5°. Ten water droplets were then dropped on the coating, using a pipette, and the number of pinned droplets was counted.

### Characterization of Particles

The particle size distribution of the primary particles was measured by dynamic light scattering using a Zetasizer Nano (Malvern Panalytical). Whereas the particle size distributions of the SPs were measured by laser diffraction using a Mastersizer (Mastersizer 2000/Hydro 2000S, Malvern Panalytical). The span of the particle size distribution was calculated using Equation 1, where *d*10, *d*50, and *d*90 are 10th, 50th, and 90th percentiles obtained from the particle size distribution.^[^ ^70^ ^]^

(1) $$span=\frac{d90-d10}{d50}$$

### Characterization of Surfaces

The particles and the coatings were analyzed by scanning electron microscopy (Gemini SEM 500 by ZEISS) using a SE2 detector, an acceleration voltage of 1 keV, and a 15 µm aperture at different magnifications. Additionally, the coatings were analyzed by light microscopy (Ergolux, Leitz with a DCC3260C microscope camera from Thorlabs).

### Characterization of Mechanical Stability

The coating stability was analyzed by a tape peel test and an abrasion test. The tape peel test was performed by applying an adhesive tape (Scotch Magic by 3M) to the coating, ensuring no air bubble entrapment under the tape and peeling it off horizontally, as shown in Figure S5a (Supporting Information). For the abrasion test, a Kimwipe tissue folded four times lengthwise was used as the abrasive medium with a weight of 100 g on top and pulled it horizontally over the coating, as illustrated in Figure S5h (Supporting Information). Multiple cycles were performed, and repellent properties were analyzed after each cycle for the first five cycles, then after the 10th, 15th, and 20th cycle.

The robustness of the coatings was tested against harsh liquids by preparing an acidic solution of 0.1 m HCl (pH 1, Hydrochloric acid, 35%, VWR) and a basic solution of 1 m NaOH (pH 14, Sodium hydroxide, ≥99%, Sigma–Aldrich).

### Statistical Analysis

The presented contact angle values are a mean of at least 5 different measurements, and the contact angle hysteresis values are a mean of at least 3 different measurements on the respective coatings. The error bars represent the standard deviation of these measurements. The pinning fraction is calculated from dropping ten different droplets on the coatings. The roll‐off angle is measured once for each case by using three droplets placed on the coatings, as described above.

## Conflict of Interest

The authors declare no conflict of interest.

## Author Contributions

U.S. designed and realized the experiments, performed synthesis, fabrication, characterization, analysis, interpretation, and conceptualization, and wrote the original draft. T.W. contributed to the design, interpretation, and conceptualization. C.W. performed synthesis, characterization, and analysis under the supervision of U.S. and T.W. L.S. performed fabrication and characterization under the supervision of U.S. N.V. acquired the funding, performed conceptualization and supervision. All the authors reviewed and edited the final manuscript.

## Supporting information



Supporting Information

Supplementary Video 1

Supplementary Video 2

Supplementary Video 3

Supplementary Video 4

## Data Availability

The data that supports the findings of this study is openly available on Zenodo at: [https://doi.org/10.5281/zenodo.16054027], reference number [16054027].
