# Supplementary material for: A Supraparticle‐Based Approach to Robust Biomimetic Superhydrophobic Coatings
Source: Small. 2025 Sep 5;21(42):e05850. doi: 10.1002/smll.202505850 (PMC12548018; doi:10.1002/smll.202505850)
Supplement: Supplementary file 1 — Supporting Information [file SMLL-21-e05850-s003.pdf]

NANO · MICRO
small

Supporting Information

for *Small*, DOI 10.1002/smll.202505850

A Supraparticle-Based Approach to Robust Biomimetic Superhydrophobic Coatings

*Umair Sultan, Teresa Walter, Christian Wachter, Leon Swart and Nicolas Vogel**

Supporting Information

A supraparticle-based approach to robust biomimetic superhydrophobic coatings

*Umair Sultan[1], Teresa Walter[1], Christian Wachter[1], Leon Swart[1], Nicolas Vogel[1]**

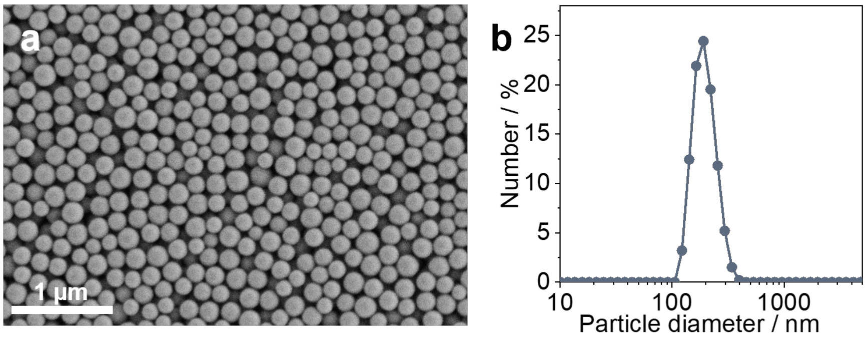

Figure S1. Silica primary particles. (a) SEM image of the 200 nm diameter silica primary particles. (b) Particle size distribution of the silica primary particles.

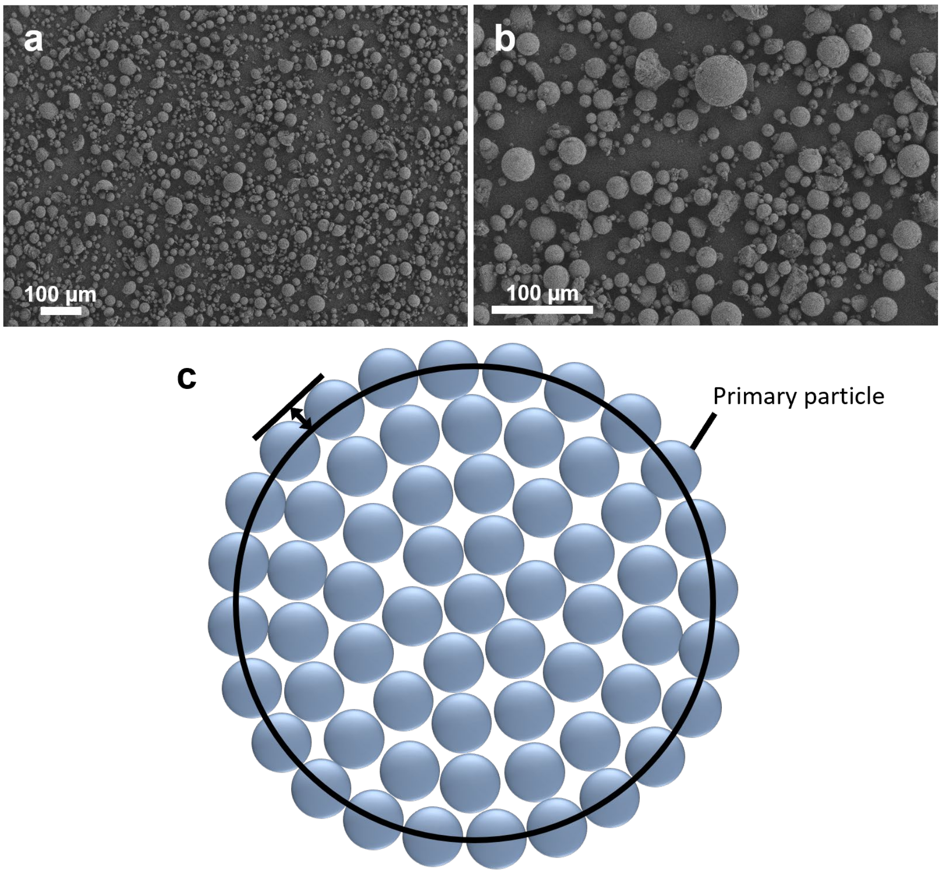

Figure S2. Spray-dried supraparticles (SPs). (a, b) Low magnification SEM images of the SP powder used to fabricate the superhydrophobic repellent coatings. (c) Schematic illustration of the SP cross-section, highlighting its surface roughness that is approximately equivalent to the radius of the primary particles.

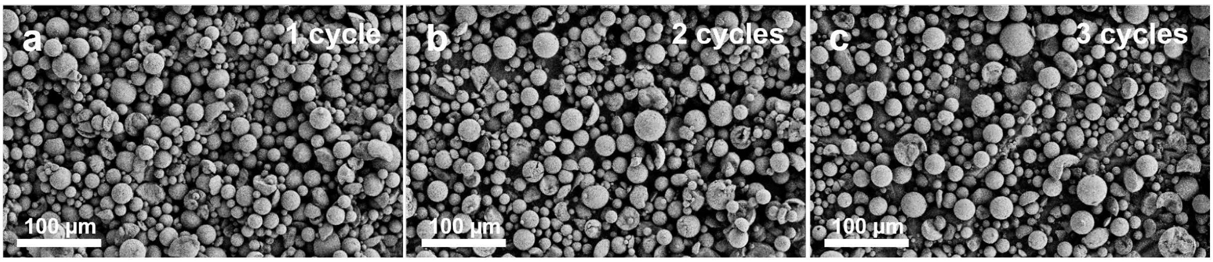

Figure S3. Fabrication of the supraparticle-based repellent coatings using spray coating. (a-c) Top-view SEM images of the fabricated coatings using one, two and three cycles of spray coating, respectively. Multiple cycles of spray coating do not have any significant influence on the supraparticle density in the coating.

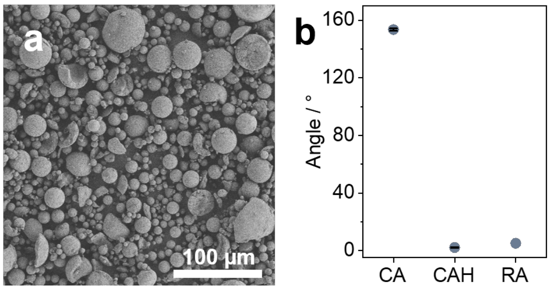

Figure S4. Supraparticle-based superhydrophobic coatings fabricated using dip coating method with polydimethylsiloxane (PDMS) as primer layer. (a) Top-view SEM images of the fabricated coatings. (b) Static contact angle (CA), contact angle hysteresis, (CAH) and roll-off angle (RA) of the fabricated coatings.

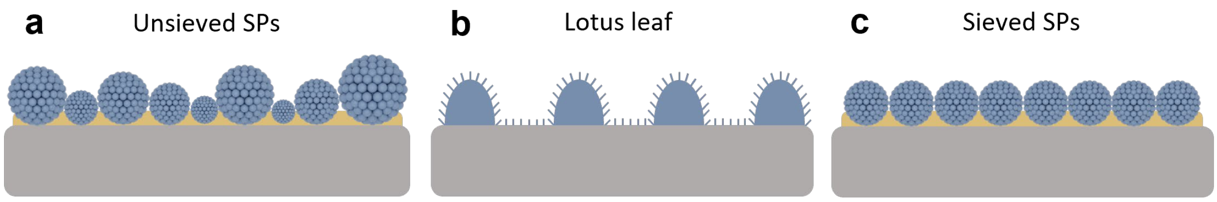

Figure S5. Schematic illustration of the cross-section of repellent surfaces. (a) Repellent coatings formed using unsieved supraparticles (SPs). (b) Hierarchical surface structure of the lotus leaf. (c) Superhydrophobic coatings fabricated using sieved SPs. The surface structure of coatings fabricated using unsieved SPs closely resembles the lotus leaf surface.

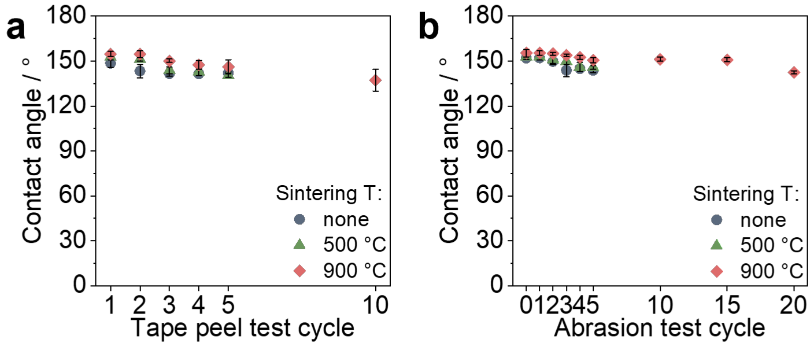

Figure S6. Mechanical stability of coatings prepared using polydimethylsiloxane (PDMS) primer layer and unsintered and sintered supraparticles. (a, b) Static contact angle (CA) of the coatings subjected to multiple cycles of (a) tape peel test and (b) abrasion test.

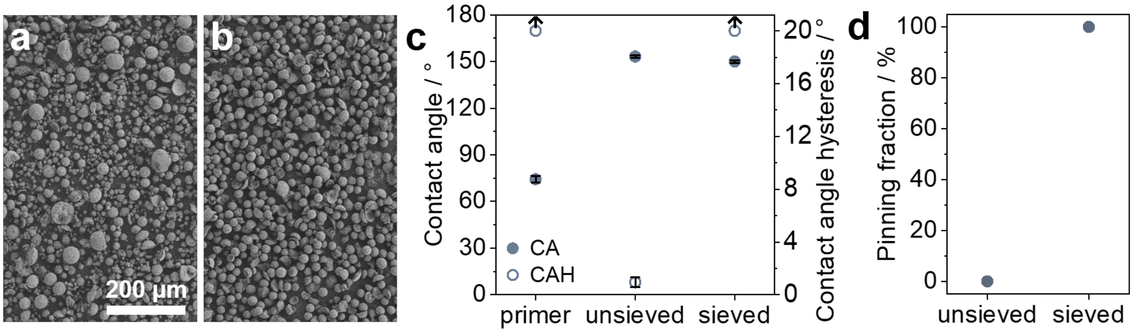

Figure S7. Supraparticle-based superhydrophobic coatings fabricated using polyurethane as primer layer. (a, b) Top-view SEM images of the coatings fabricated from unsieved and sieved supraparticles, respectively. (d) Static contact angle (CA) and contact angle hysteresis (CAH) of the polyurethane primer layer and the coatings fabricated using unsieved and sieved supraparticles. (e) Pinning fraction (from 10 water droplets) of each of the fabricated coatings. The coatings exhibit similar water repellent properties as the ones fabricated using polydimethylsiloxane primer layer.

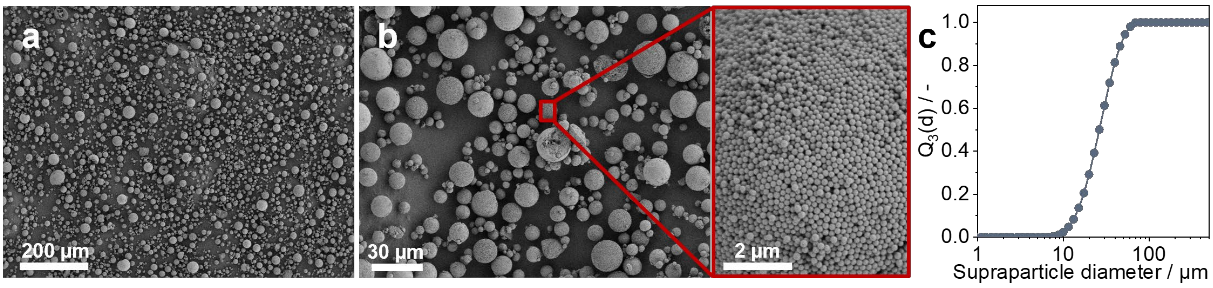

Figure S8. Fabrication of supraparticles (SPs) containing water glass binder. (a, b) Low and high magnification SEM images of the binder-containing spray-dried SPs. The surface roughness can be observed in the high-magnification inset. (c) Particle size distribution of the binder-containing spray-dried SPs.

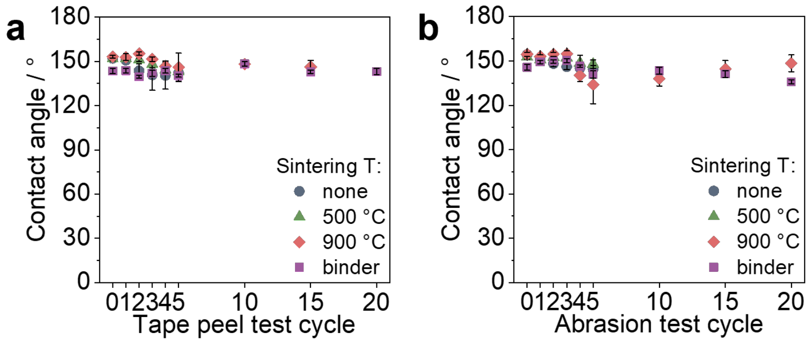

Figure S9. Mechanical stability of coatings prepared with a polyurethane (PUR) primer layer and unsintered and sintered supraparticles. (a, b) Static contact angle (CA) of the coatings subjected to multiple cycles of (a) tape peel test and (b) abrasion test.

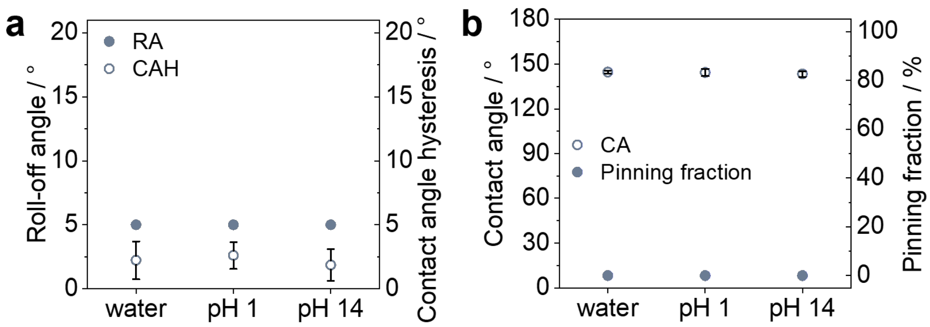

Figure S10. Testing of superhydrophobic coatings prepared with a polyurethane (PUR) primer layer and binder-containing supraparticles by exposing them to acidic and basic liquids. (a) Roll-off angle (RA) and contact angle hysteresis (CAH) of the coatings. (b) Static contact angle (CA) and pinning fraction of the coatings.

Table S1. Mechanical stability of the fabricated superhydrophobic coatings.

Primer layer	Supraparticle type	Tape peel test cycles until failure	Abrasion test cycles until failure
PDMS	Unsintered	1 cycle	2 cycles
PDMS	Sintered at 500 °C	2 cycles	3 cycles
PDMS	Sintered at 900 °C	5 cycles	5 cycles
PUR	Unsintered	2 cycles	2 cycles
PUR	Sintered at 500 °C	3 cycles	4 cycles
PUR	Sintered at 900 °C	4 cycles	5 cycles
PUR	Water glass binder and sintered at 900 °C	25 cycles	15 cycles

Supplementary Videos Captions

Video S1. Droplets of ink rolling down the superhydrophobic repellent coating on glass substrate (tilting angle=5°) fabricated using non-sintered supraparticles and spray coating method.

Video S2. Superhydrophobic coating fabricated using binder-containing supraparticles being subjected to a stream of water. The water is completely repelled by the coatings.

Video S3. Superhydrophobic coating fabricated using binder-containing supraparticles being subjected to a stream of acidic solution (pH 1). The acidic solution is completely repelled by the coatings.

Video S4. Demonstration of the self-cleaning effect. Superhydrophobic coating fabricated using binder-containing supraparticles covered with fluorescent high-density polyethylene powder. The powder is easily removed by washing with water.
